# Biopsychosocial Approaches for the Management of Female Chronic Pelvic Pain: A Systematic Review

**DOI:** 10.1111/1471-0528.17987

**Published:** 2024-10-27

**Authors:** Selina Johnson, Alison Bradshaw, Rebecca Bresnahan, Emma Evans, Katie Herron, Dharani K. Hapangama

**Affiliations:** ^1^ Pain Management Department Walton Centre NHS Foundation Trust Liverpool UK; ^2^ Department of Women's and Children's Health, Institute of Life Course and Medical Sciences University of Liverpool Liverpool UK; ^3^ Liverpool Reviews and Implementation Group, Institute of Population Health University of Liverpool Liverpool UK; ^4^ Nuffield Department of Women's and Reproductive Health University of Oxford Oxford UK

**Keywords:** biopsychosocial, chronic pelvic pain, female, outcome, review

## Abstract

**Background/Objective:**

Current guidelines recommend biopsychosocial‐informed treatment for chronic pelvic pain (CPP). The objective of this systematic review was to describe the available biopsychosocial approaches for the treatment of CPP, and the outcomes reported, to understand how guideline‐recommended treatments can be applied.

**Search Strategy:**

MEDLINE, CINAHL, PsycINFO, EMBASE, Emcare, AMED and Cochrane trial registries were searched (inception to 17 November 2023).

**Selection Criteria:**

CPP Studies in women where the principal treatment modality was a biopsychosocial approach were included. Prospero registration: CRD42022374256.

**Data Collection/Analysis:**

Data extraction included study setting, population, study design, intervention characteristics and outcome measures and is described via a narrative synthesis.

**Results:**

The review included 14 RCTs (871 patients) and identified four broad intervention categories (Acceptance Commitment Therapy *n* = 2, Cognitive Behavioural Therapy *n* = 6, Mindfulness‐based approaches *n* = 2, and Physiotherapy‐based interventions *n* = 4). Pain science education (PSE) and, exposure/engagement with valued activity were recognised as important aspects of treatment regardless of intervention type. The most utilised outcomes were pain reduction and emotional functioning, with all studies reporting improvements in these domains. Heterogeneity in outcomes prevented efficacy comparison. High risk of bias was identified in six studies (1/4 physiotherapy‐based approaches, 2/6 CBT, 1/2 ACT and 2/2 mindfulness‐based interventions).

**Conclusions:**

CBT and ACT‐based biopsychosocial approaches were found effective in reducing pain and improving psychometric outcomes for CPP. Evaluation indicated PSE, and exposure/engagement in valued activity are important components of biopsychosocial management. Outcome heterogeneity needs to be addressed in future trials.

## 1 Introduction

Chronic pelvic pain (CPP) is chronic or persistent pain perceived in structures related to the pelvis [1]. Chronic pain is pain that persists or recurs for longer than 3 months [2, 3].

CPP is a common presentation in UK primary care, with incidence of 38 per 1000 women affected annually – a rate comparable to those of asthma (37 per 1000) and back pain (41 per 1000) [3].

CPP can be extremely debilitating and can negatively impact social and occupational abilities, emotional well‐being and relationships [1]. Additionally, CPP is commonly associated with difficulties in sexual relations and bladder and bowel dysfunction [1, 4]. Living with CPP is associated with reduced quality of life (QoL) [5, 6].

The annual costs of CPP to the Nation Health Service have been estimated at approximately £326 million [7]. This expenditure is similar to or higher than the annual medical costs per person of other chronic diseases such as heart disease and diabetes [8]. Whilst annual indirect costs associated with loss of work productivity are thought to double this figure [8]. It is estimated that only about a third of women seek medical help; therefore, further uncaptured costs associated with pain impact and reduced QoL are likely [9].

Current understanding of chronic pain recognises the role of complex pain mechanisms and psychological, behavioural and social factors that can drive and perpetuate pain [10]. Consequently, biopsychosocial approaches that address these factors are recommended for chronic pain conditions to improve pain and QoL [1, 11]. Recommendations within current guidelines are nonspecific regarding how biopsychosocial approaches for CPP should be applied and which approaches are most effective and therefore provide limited direction for clinicians [12, 13, 14, 15, 16, 17, 18]. Consequently, the literature describes a variety of approaches for CPP, including both single‐discipline and multidisciplinary models. For clinicians and persons living with CPP, it is important to understand the nature and benefits of different approaches to inform treatment selection and optimise care.

This systematic literature review aims to identify currently used biopsychosocial approaches for female CPP, to identify what efficacy outcomes are used and to evaluate the quality of supporting evidence.

## 2 Methods

A systematic review was conducted based on the Preferred Reporting Items for Systematic Reviews and Meta‐Analyses (PRISMA) guidelines [19]. The review protocol was prospectively registered: PROSPERO: CRD42022374256.

### 2.1 Search Strategy and Study Selection

MEDLINE, CINAHL, PsycINFO, EMBASE, Emcare, AMED and Cochrane trial registries were searched from inception until 17 November 2022 (initial search date and then updated 17/11/23). Included abstracts proceeded to a full‐text review. All abstracts and full texts were screened using predetermined inclusion/exclusion criteria (Table 1) independently by two authors using Rayyan software (SJ/AB), with any disagreements settled by a third author (RB). For example, search strategy see Appendix S1.

**TABLE 1:** Inclusion/exclusion criteria.

	Include	Exclude
Population	Primary diagnosis of chronic pelvic pain, chronic pelvic girdle pain, endometriosis pain syndrome or pelvic pain duration ≥ 6 months Women aged 18 years and over (here we use the term women to refer to anyone assigned female at birth).	Acute pain defined < 6 months duration. Subjects/populations where the primary pain complaint is NOT CPP
Intervention	Biopsychosocial approach cited as the principal modality. Recognised biopsychosocial approaches (e.g., Acceptance commitment therapy (ACT) and Cognitive behavioural therapy). Treatments delivered within a biopsychosocial framework. Framework had to include supported understanding of the wider impact of pain (mood and cognitions), CPP education, and strategies to manage social/activity impact.	Medical, surgical, or pharmacological interventions.
Comparator	Any or none	n/a
Outcomes	Outcomes measures of efficacy	No reported outcomes relating to efficacy or change
Study design	RCT	Sample size of < 10 – as these will limit evaluation of effect. Abstracts – due to insufficient detail to assess study quality.
Language	English	Non‐English

### 2.2 Quality Assessment

Quality assessment was independently conducted by two authors (SJ and AB) using the Cochrane risk of bias tool (RoB 2) [20], and any discrepancies resolved by a third author (RB).

### 2.3 Data Extraction

Data on study setting, population, study design, intervention characteristics, outcome measures, timing of outcomes and study findings were extracted from the eligible papers.

### 2.4 Narrative Synthesis

The findings were summarised with a narrative synthesis. Studies are described within four broad intervention categories, determined by authors based on the main underpinning theory of the intervention. They included, (i) physiotherapy‐based interventions described as physical therapy‐based treatment, (ii) Cognitive Behavioural Therapy (CBT), (iii) Acceptance Commitment Therapy (ACT) informed interventions that included and described six core processes of acceptance, diffusion, contact with the present moment, self as context, values and committed action and (iv) mindfulness‐based interventions that described mindfulness based treatment but did not include all ACT core processes. Previous work has highlighted outcome heterogeneity in CPP trials and called for the development of a core outcome set for CPP [21]. Working towards obtaining an international consensus on outcomes for CPP trials The International Collaboration for Harmonising Outcomes, Research, Standards in Urogynaecology (CHORUS) has recommended the following outcome domains be considered (i) Pain, (ii) Life impact (QoL, emotional functioning, physical functioning), (iii) Clinical effectiveness (efficacy, satisfaction, cost‐effectiveness, return to Activities of Daily Life (ADLS)) and (iv) Adverse events [22]. We have reviewed the literature concerning the inclusion of these outcome domains. We also assessed how biopsychosocial impact was measured and considered the following domains of ‘life impact’, (i) QoL, (ii) psychological function, (iii) objective measures of physical function, (iv) bladder and bowel, (v) sexual function and (vi) overall activity functionality (i.e., work and ADLS). Each study was given a score of 6 reflecting the number of these domains it included (see outcomes).

For all full‐text RCTs, where standard deviation and mean changes were not reported, authors were contacted and given 3 weeks to respond, receiving no responses. There was considerable heterogeneity in research outcomes, types of control and treatment dosage, therefore, a narrative synthesis of results presented.

## 3 Results

**FIGURE 1:**
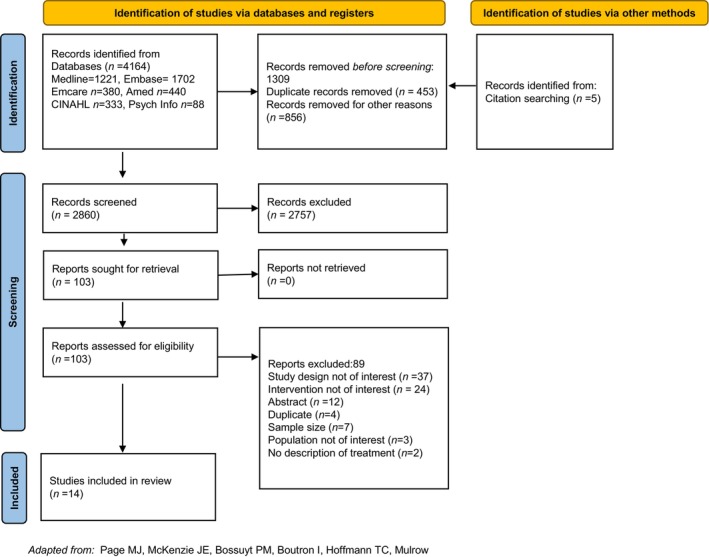
Prisma diagram, a flowchart of inclusion.

From 4169 citations, 14 RCTs (Table S1) were included in the systematic review (Figure 1).

### 3.1 Quality Assessment

There were concerns with eight studies (4/4 physio‐based, 3/6 CBT and 1/2 ACT) and six had a high risk of bias (1/4 physio‐based approaches, 2/6 CBT, 1/2 ACT and 2/2 mindfulness‐based interventions) (Figure S1) mostly related to deviations from the intended intervention and selection of reported results (see S2).

### 3.2 Biopsychosocial Approaches (Tables S1 and S2)

#### 3.2.1 Physiotherapy‐Based Interventions

Four included studies reported statistically significant superiority for physiotherapy in comparison to other treatments [23, 24, 25, 26]. Core components across all four studies included education (information about CPP, physical activity, fear of movement, beliefs, active lifestyle and behavioural advice), value‐orientated goal setting, graded exercises and activity management strategies.

Three studies (conducted by the same research group) delivered 1:1 physiotherapy, with the main content differences being the emphasis or focus of goal setting. Rodrıguez‐Torres [25], described an ‘individualised comprehensive rehabilitation program’ (ICPR) (*n* = 38) where goals were graded to avoid provoking pain increases. Ariza‐Mateos (*N* = 44) [23] described ‘The Cumulative‐Complexity Model’ where goals were developed factoring in patient‐perceived workload‐capacity difficulties (e.g., energy, time and functional limitations). In the third study (*N* = 49), goals were informed by graded exposure principles (GET) and feared activities were ranked and progressed as fear and pain reduced [24]. GET was either combined with manual therapy (MT) or delivered as a standalone treatment. All three studies compared treatment (6–8 weeks) to an educational (leaflet) control comprising the same educational topics.

The fourth study described a 10‐day group‐based ‘multimodal physical therapy’ programme compared to women's health physiotherapy (WH‐PT) (*N* = 51) [26]. This included further educational sessions on pain neurobiology, an overview of ACT, nutrition, and hydrotherapy. To ‘reflect standard practice’ WH‐PT was determined by the treating therapist (no description of training). Within WH‐PT 30% received pelvic floor training combined with general exercises, and 50% received MT. Whilst this study included a session on ACT, it did not specifically reference the core processes and therefore, was grouped as a physiotherapy‐based intervention [26].

Three of the four studies included post‐treatment follow‐up (FU) with maintenance of effect at 3 months for the ICPR and GET treatments [24, 25], and 12 months for the ‘multimodal physical therapy’ [26].

#### 3.2.2 Cognitive Behavioural Therapy (CBT)

Six included RCTs applied CBT interventions for provoked vestibulodynia (PVD) [27, 28, 29, 30, 31, 32]. CBT in all studies included psychological & PVD education, cognitive restructuring, cognitive diffusion, relaxation components, progressive muscle relaxation and reconceptualisation of PVD.

##### 3.2.2.1 CBT Delivered on a 1:1 Basis

Two studies compared the efficacy of a 1:1 weekly CBT session against different comparators [27, 31].

Goldfinger et al. [31] (*N* = 20) compared CBT to ‘physiotherapy’ (8 weeks) both were associated with clinically meaningful improvement post‐treatment and 6‐month FU. ‘Physiotherapy’ included pain science education, pelvic floor exercises and dilators for progressive vaginal penetration (also included in the CBT arm) in addition to surface electromyography (EMG), hip stretches, deep breathing and relaxation.

Masheb et al. [27] (*N* = 50) found CBT was significantly superior to non‐behavioural psychotherapy (10 weeks) at 6‐ and 12‐month FU. Non‐behavioural psychotherapy involves assisting patients to express their feelings whilst not suggesting how they should change.

##### 3.2.2.2 Group CBT

Two studies report statistically significant superiority for group CBT [28, 32], whereas two studies report equal efficacy for group CBT and comparator groups (MBCT [30], CBT, surface electromyography (EMG) and vestibulectomy [29]).

Bergeron et al. [28] (*n* = 108), found couples‐based CBT (CCBT) superior to topical lidocaine in all areas except pain reduction. Specific to CCBT was the inclusion of ‘expansion of sexual repertoire’, and exercises to improve pain and sexuality‐related interactions.

Guillet et al. [32] (*N* = 31) found Mindfulness CBT (MCBT) superior to an educational video control. MCBT described CBT with the addition of mindfulness meditations and exercises. Video topics were vestibule skin pain, sore pelvic muscles and psychological dysfunction.

Brotto et al. [30] (*N* = 130) reported comparable efficacy for MBCT compared to CBT.

A further study by Bergeron et al. (*n* = 78) found comparable efficacy for CBT, surface electromyography (EMG) and vestibulectomy (surgical removal of painful tissue) in all areas except pain reduction (*N* = 78) [29]. Vestibulectomy was associated with greater pain reduction and higher attrition (27% attrition compared to 4% for other groups).

Across all studies improvements were maintained at 6‐month FU following 8–12 weeks of treatment.

#### 3.2.3 Acceptance and Commitment Therapy (ACT)

Two studies examined ACT [33, 34], finding them superior to waiting list control (WLC) [33, 34].

Hess Engstrom et al. described an ACT internet‐based intervention (*n* = 99) for women with PVD [35], involving six online modules. Despite high attrition rates, treatment benefits were maintained at 10 months.

Hansen et al. evaluated MY‐ ENDO (group‐based mindfulness and ACT), ‘non‐specific’ psychology and WLC (*n* = 58) [33]. Both interventions were found to be equally effective post‐treatment and at 12‐week FU. MY‐ENDO consisted of a manualised programme of weekly group sessions and Yoga exercises (10 weeks). The ‘non‐specific psychology’ used the same format with the removal of all aspects specific to mindfulness & ACT, and yoga was replaced by relaxation to music.

#### 3.2.4 Mindfulness Informed Approaches

In addition to studies that combined mindfulness with either CBT or ACT, two further studies were identified where the main component was mindfulness [36, 37]. Similarly to the MCBT, both studies included mindfulness exercises, meditations, psycho‐pain education and activity management.

de Moreira, Gamboa, and Pinho Oliveira [36] (*n* = 32) reported treatment superiority for a ‘brief group‐based mindfulness‐based intervention’ (bMBI) compared to usual care (UC). bMBI addressed acceptance, avoidance, habits, and behaviours (4 weeks). UC consisted of hormonal therapy or analgesic medications.

Whilst comparable efficacy was found for online mindfulness‐based stress reduction (MBSR) and healthy lifestyle intervention (HL) (6‐weeks) [37]. MBSR topics included thoughts and reactions, responding, and reacting, mindful movements and journal writing. HL treatment included content on nutrition, exercise choices and target setting.

Across studies, details regarding who delivered the intervention and the level of training were often ambiguous (Table S1).

### 3.3 Outcomes Measures

There was significant heterogeneity in research outcomes, type of control and treatment intervention in addition to incomplete reporting of standard deviation and mean change data. This prevented comparative efficacy data pooling. Therefore, a narrative synthesis of outcomes is provided below (Table S3 illustrates reported outcomes for all studies), a descriptive comparison of absolute effects is described in Appendix S2.

#### 3.3.1 Pain

Improvements in pain intensity were reported by 13/14 RCTs (Table 2). Overall, all biopsychosocial interventions were associated with significant pain reductions regardless of the measure used post‐treatment. High attrition bias limits the certainty of mindfulness and internet‐based ACT results. Pain reduction at FU (beyond the treatment period) was reported in all treatment categories except for mindfulness‐based interventions.

**TABLE 2:** Pain outcomes measures across included studies.

	Pop	*n* (intervention arm)	Intervention	Outcome measure	Time reference point	Baseline	Post‐change	FU change
*Physio‐based*								
Rodrıguez‐Torres [25] 2020	CPP	19	ICPR	BPI Severity	24 h	6.01 (1.95)	2.5	0.49
Ariza‐Mateos [23] 2020	CPP	22	Cumulative‐complexity model	VAS	NS	5.39 (1.95)	3.18	
Ariza‐Mateos [24] 2019	CPP	16	GET + MT	BPI Severity	24 h	6.01 (1.95)	1.68	2.75
Nygaard [26] 2020	CPP	26	Group multimodal physio	NRS last 7 days	1 week	4.8 (2)	1.1	1.8
*CBT*								
Goldfinger [31] 2016	PVD	10	CBT 1:1	NRS intimacy	NS	5.2 (1.40)	2.6	3.1
Masheb [27] 2009	PVD	25	CBT 1:1	MPI (0–6 scale)	In moment	2.6 (1.2)	1	1.3
Bergeron [29] 2001	PVD	26	CBT group	NRS intimacy	NS	7.14 (1.53)	1.4	2.68
Bergeron [28] 2021	PVD	55	CCBT	NRS intimacy	3–6 months	6.81 (1.77)	2.11	2.36
Brotto [30] 2019	PVD	67 CBT 63 MBCT	CBT MBCT	NRS intimacy	NS	5.86 (2.13)/6.69 (1.91)	1.21/2.35	1.83/3.3
Guillet [32] 2019	PVD	14	MBCT	Tampon test	In moment	N/S	−1.33	−2.4
*ACT*								
Hess Engstrom [34] 2021	PVD	52^a^	ACT internet‐based	NRS intimacy	1 month	6.85 (2.05)	3.52	3.15
Hansen [33] 2023	CPP	20	MY‐ENDO	NRS daily diary	Daily diaries	6.11 (2.05)	NS	NS
*Mindfulness‐based*								
Moreira [36] 2022	CPP	31^a^	bMBI	NRS	1 month	8.5 [7.25, 10]	3.5	3.5
Crisp [37] 2023	CPP	21^a^	MBSR	BPI Severity	24 h	NS	NS	NS

*Note:* Figures in brackets denote standard deviation, figures in square brackets indicate Median and q1, q3.

Abbreviations: ACT, acceptance commitment therapy; bMBI, brief mindfulness‐based intervention; BPI, brief pain inventory; CBT 1:1, Individualised cognitive behavioural therapy; CCBT, couples cognitive behavioural therapy; CPP, chronic pelvic pain; GET + MT, Graded exposure and manual therapy; ICPR, Individualised comprehensive rehabilitation program; MBCT, mindfulness‐based CBT; MBSR, mindfulness‐based stress reduction therapy; MPI, McGill pain inventory; MY‐ENDO, Mindfulness and acceptance‐based therapy intervention; NRS, numerical rating scale; NS, not stated; POP, population; PVD, provoked vulvodynia; VAS, visual analogue scale.

^a^ > 20% attrition from baseline.

#### 3.3.2 Life Impact

One study [26] assessed five of the six ‘life impact’ domains that are listed in Table 3, whilst all others scored 2 or 3.

**TABLE 3:** Outcome measures related to ‘life impact’.

Outcome domain	QoL	Psychological function	Physical function	Bladder and bowel	Sexual function	Functionality (work and ADLS)	SCORE 0–6
*Physio‐based treatments*							
Rodrıguez‐Torres [25] 2020	EQ5D+		MiniBEST+ TUG+			BPI Int+, ODI+	3
Ariza‐Mateos [23] 2020	EQ5D+	CSQ−				COPM+, IPAQ+	3
Ariza‐Mateos [24] 2019		FABQ+				BPI Int+, ODI+	2
Nygaard [26] 2020	EQ5D−	TSK+ Hopkins Symptom checklist−	Mensendieck+	Continence ICIQ‐UI	Sex function Y/N		5
*Cognitive behavioural therapy (CBT) informed interventions*							
Goldfinger [31] 2016		PCS+ CSQ+			FSFI+		2
Masheb [27] 2009		PASS+ BDI−			FSFI+		2
Bergeron [29] 2001		BSI− global severity index+			Sexual history form+, DSFI+		2
Bergeron [28] 2021		PASS+ PCS+			FSFI+ FSDS+		2
Brotto [30] 2019		PCS+ PVAQ+ CPAQ+			FSFI+ FSDR+		2
Guillet [32] 2019		PCS+ GAD‐7+ BDI+			FSDS+ FSFI+		2
*Acceptance commitment therapy (ACT) interventions that included and described acceptance, diffusion, contact with the present moment, self as context, values and committed action*							
Hess Engstrom [34] 2021		CPAQ+			Impact %		2
Hansen [33] 2023	EHP‐30−, but + for control/powerless/emotional wellbeing & social subscales	CPAQ+				WAI+	3
*Mindfulness‐based interventions that described mindfulness‐based treatment but did not include all ACT core processes*							
Moreira [36] 2022	SF‐36−	SF‐36 Mental health subscale					2
Crisp [37] 2023		PHQ‐9+				BPI Int+	2

*Note:* − denotes no significant change; + denotes significant improvement.

Abbreviations: BDI, Beck Depression Inventory; BPI Int, brief pain inventory interference subscale; BSI, Brief Symptom Inventory; COPM, Canadian occupational performance measure; CPAQ, Chronic Pain Acceptance Questionnaire; CSQ, Cognitive Style Questionnaire; DSFI, Derogatis Sexual Functioning Questionnaire; EHP‐30, Endometriosis Health Questionnaire; EQ5D, EQ5D‐5L; FABQ, Fear Avoidance Beliefs Questionnaire; FSDR, Female Sexual Distress Scale Revised; FSDS, Female Sexual Distress Scale; FSFI, Female Sexual Function index; GAD‐7, General Anxiety Disorder Assessment; ICIQ‐UI, International Consultation on Continence Questionnaire; ODI, Owestry disability index; PASS, Pain Anxiety Symptom Scale; PCS, Pain Catastrophising Questionnaire; PHQ‐9, Pain Health Questionnaire; PSS, Patient Symptom Scale; PVAQ, Pain Vigilance and Awareness Questionnaire; QoL, quality of life; TSK, Tampa Scale of Kinesiophobia; TUG, Timed Get Up and Go; WAI, workability Index.

##### 3.3.2.1 QoL

Five studies measured QoL, with three reporting significant improvements [23, 25, 26, 33, 36]. Total score improvement was reported in 2 physiotherapy‐based intervention studies [23, 25]. Whilst subscale improvement was reported for ACT‐based MY‐ENDO (subscales of control/powerless/emotional wellbeing and social) [33] and bMBI (mental health subscale) [36].

##### 3.3.2.2 Psychological Function

Thirteen studies measured change in psychological function [23, 24, 26, 27, 28, 29, 30, 31, 32, 33, 34, 36, 37]. Ten of the thirteen reported significant improvements in psychological outcomes (Two physiotherapy‐based studies that included GET, all six CBT studies and both ACT studies). The physio‐based studies included psychological outcomes that explored the fear of movement/avoidance but no other measures of psychological function. The CBT studies included a range of outcomes that [24], including the Pain Catastrophising Scale, Pain Anxiety Symptom Inventory, Cognitive Style Questionnaire, Brief Symptom Inventory, Chronic Pain Acceptance Questionnaire (CPAQ), Pain Vigilance and Awareness Questionnaire, Beck Depression Inventory and General Anxiety Disorder Assessment [27, 28, 29, 30, 31, 32]. The two ACT‐based studies assessed pain acceptance (CPAQ) only [33, 34].

##### 3.3.2.3 Bladder and Bowel Function

One study reported improvement of 0.4 (*p* = 0.0370) for ‘multimodal physiotherapy’ and no change in WH‐PT using the International Consultation on Incontinence Questionnaire‐Urinary Incontinence (ICIQ‐UI) (score 0–21, minimum clinically important difference (MCID) for non‐surgical interventions = 4) [26, 38].

##### 3.3.2.4 Objective Measures of Physical Function

Two physiotherapy‐based interventions included objective measures of function which demonstrated significant improvements in posture and dynamic movements of sit‐stand and gait [25, 26].

##### 3.3.2.5 Sexual Function

Only the six CBT studies used validated measures of sexual function; five different outcomes were used (FSFI = Female Sexual Function Index, DSFI=Derogatis Sexual Functioning Questionnaire, FSDR = Female Sexual Distress Scale‐Revised, FSDS = Female Sexual Distress Scale) [27, 28, 29, 30, 31, 32]. Five studies used the Female Sexual Function Index (FSFI). In all cases, CBT was associated with significant improvement in sexual function.

##### 3.3.2.6 Overall Functionality

Significant improvement was reported in five studies using different generic pain rather than disease‐specific functionality outcome measures [23, 24, 25, 33, 37]. Three studies (two physiotherapy‐based and one mindfulness‐based) used the Brief Pain Inventory interference subscale (BPI Int) [24, 25, 37]. Two physiotherapy‐based studies used the Oswestry Disability Index (ODI) [24, 25]. One physiotherapy‐based study included The Canadian Occupational Performance Measure, which allowed patients to independently select specific areas of impact relevant to them.

#### 3.3.3 Cost Effectiveness

No study considered cost‐effectiveness.

#### 3.3.4 Adverse Events (AEs)

No study distinguished between serious and non‐serious AEs. Four studies only referred to AEs stating ‘no adverse events’ in response to treatments (3 physio‐based and 1 CBT) [23, 24, 26, 31] but provided no further information to understand how this was assessed [28, 29].

## 4 Discussion

### 4.1 Main Findings

This review identified biopsychosocial approaches involving four broad intervention categories for managing CPP in women (physio‐based approaches, CBT, ACT and mindfulness‐based interventions). Despite differences, the interventions shared the following common themes pain science education (PSE), exposure to and engagement with valued activities. Pain reduction and psychometric outcomes were the most assessed outcomes, with consistent benefits reported despite variations in outcome selection. Six studies showed a high risk of bias (1/4 physiotherapy‐based, 2/6 CBT, 1/2 ACT, and 2/2 mindfulness‐based interventions).

### 4.2 Interpretation

#### 4.2.1 Intervention Themes

PSE is considered central to successful pain management and was included in all reviewed RCTs [39, 40, 41]. Recent work exploring PSE for persons with CPP identifies key concepts of validation, understanding how pain works, the influence of different factors and the potential for the pain to change as important [41]. Within this review, PSE that appeared to address these themes positively affected pain and catastrophising but had minimal effect on other parameters when delivered in isolation [23, 24, 25, 32]. Results highlight that PSE when combined with behavioural and cognitive therapy positively affects parameters of mood and function. This illustrates that human behaviour is shaped by physical and psychological information and PSE is complex requiring both information and practical experience/opportunities to explore and challenge understanding [42, 43].

#### 4.2.2 Exposure to Activity

All studies included various behavioural change techniques to modify activity engagement with pelvic floor therapy (PFT) commonly included to support these processes. Pelvic floor dysfunction is frequently associated with CPP but there is little consensus on how ‘dysfunction’ is defined and the specific role of PFT [44]. Recent evidence highlights there is limited high‐quality evidence to support PFT [45, 46, 47]. All included CBT studies (reporting gains in sexual function) [27, 28, 29, 30, 31, 32] and two physio‐based studies included forms of PFT aimed at desensitisation and relaxation of the pelvic floor [24, 26] Two studies compared PFT delivered within the context of a biopsychosocial framework to PFT as an isolated treatment [24, 26]. Greater efficacy was seen for PFT delivered in the context of a biopsychosocial framework [27, 31]. Results suggest PFT may be an important aspect of CPP treatment, but it should be evaluated holistically as part of a broader biopsychosocial approach [17].

#### 4.2.3 Engagement in Valued Activity

Value‐driven goals are linked to higher acceptance and self‐efficacy in chronic pain [48, 49]. Ten of the 14 studies included value‐based goal setting, highlighting its importance in treatment. Physio‐based studies found combining value‐driven goals with graded exposure improved outcomes [23, 24, 25]. Graded exposure is an established theory‐driven treatment that aims to improve functioning by exposing patients to feared and avoided activities [50, 51]. Recent studies have identified exposure and engagement in valued activity as a key behavioural change process for patients with chronic pain [42, 43, 52]. Future work exploring this in CPP is recommended.

Partner involvement is for many an important area of value and potentially an underexplored area in CPP research [53]. One study incorporated couples therapy to explore interactions with sexual partners [28]. Important contextual factors such as broader relationship difficulties, sexual orientation and gender identity were not described and require more attention in future research to support clinical treatment [53].

##### 4.2.3.1 ACT and CBT

ACT and CBT are established chronic pain treatments [54], but evidence in CPP is less robust [55]. Our review found both CBT and ACT effective for CPP. When ACT was compared to ‘non‐specific psychology,’ both approaches were equally effective. The ‘non‐specific psychology’ components resembled CBT components in other studies, suggesting no clear superiority between CBT and ACT [33]. More research is needed to compare the effectiveness of these two approaches and better understand their respective benefits.

#### 4.2.4 Outcomes

All studies reported improvements in pain and psychometric outcomes, but heterogeneity in outcome measures made comparisons difficult. Furthermore, available outcomes did not comprehensively capture all potentially relevant CPP life impact domains. For example, dyspareunia affects over 58% of women with CPP, with more than 51% avoiding intimacy [56]. Sexual dysfunction is closely linked to poor QoL, and therefore an important issue for many women [57, 58], and was assessed in only six of 14 studies. Additionally, no study evaluated cost‐effectiveness, a key consideration for informing clinical practice [59]. These findings support previous work highlighting inconsistency in outcome collection that calls for a core outcome set (COS) for CPP [21, 22]. The main goal of COS is to promote more relevant and self‐consistent bodies of research evidence [60]. Extensive COS work has been undertaken for various conditions including work by the Initiative on Methods, Measurement, and Pain Assessment in Clinical Trials (IMMPACT) supporting chronic pain COS [61, 62, 63]. The growing trend to develop COS has the potential to lead to confusion and unintended redundancies [60]. Better alignment and collaboration with existing chronic pain COS could further increase the impact of findings and aid the understanding of potential mechanisms of change [60].

##### 4.2.4.1 Group vs. Individualised

Both group‐based and individualised treatments were included in the studies. In clinical practice, the decision to provide group/individual treatment is influenced by both service factors (e.g., what's available/waiting times), and individual needs (e.g., relevant processes of change, outcomes of interest, environmental and personal factors that may influence engagement/participation). Although the studies did not discuss these factors, they are important to consider in evaluating and implementing treatment approaches [64, 65].

### 4.3 Strengths and Limitations

This is the first comprehensive review of guideline‐endorsed biopsychosocial treatment approaches. The search strategy was developed by CPP clinicians and an academic librarian; the protocol was prospectively registered pre‐search [66]; all abstracts and full texts were independently screened by two reviewers to reduce bias and minimise errors, with a third reviewer resolving any conflicts.

A limitation of identified CPP studies was the heterogeneity in outcomes, timeframes, interventions, underlying CPP diagnosis, and controls prevented comparative quantitative data pooling. Nine out of 14 studies had concerns about bias, and six were deemed high bias.

Another limitation was the lack of time reference points for outcomes. For example, pain, pain impact and consequently treatment responses may vary considerably depending on menstrual cycles. Future studies should better define outcome time reference points so this can be clearly understood.

Additionally, the review only included published RCTs, excluding observational and cohort studies. that may provide further clinically relevant insights [67].

Variability in follow‐up (FU) periods also limited the ability to assess long‐term effects, with attrition remaining a challenge for studies with longer FU, as seen by both the Nygaard and Hess Engstrom studies [26, 34]. Future studies need to consider how attrition bias can be managed in the context of FU.

Whilst biopsychosocial treatment appears to be effective, the review illustrates a range of approaches which contribute to disparities in care. Stratifying patients based on the risk of poor outcomes and then matching patients to appropriate treatments has been found clinically and cost‐effective for back pain [68, 69]. For CPP, this requires a better understanding of outcomes throughout the pathway but may help to reduce disparities and improve outcomes.

## 5 Conclusion

This systematic review of RCTs shows that physiotherapy‐based, CBT and ACT‐based biopsychosocial interventions are effective in reducing pain and improving psychometric outcomes for CPP. PSE and engagement in valued activities are key treatment components. However, inconsistencies in outcome selection suggest the need for better alignment with core outcome sets for chronic pain research.

## Author Contributions

S.J., A.B., and D.K.H. conceived the study. S.J., A.B., and R.B. conducted searches and analyses. K.H., A.B., D.K.H., E.E. and S.J. reviewed the analysis to inform the paper's main themes. S.J. wrote the paper and A.B., K.H., R.B., D.K.H. and E.E. all contributed to the write‐up and refinement of the submitted paper.

## Conflicts of Interest

S.J., A.B., K.H., R.B. and E.E. have no competing interests. D.K.H. is supported by the Wellbeing of Women (RG2137) and MRC (MR/V007238/1). D.K.H. has received payment for presentations from Theramex and Gideon Richter.

## Supporting information


**Table S1.** Biopsychosocial approaches used for chronic pelvic pain.
**Table S2.** Characteristics of included studies.
**Table S3.** Included studies reported outcome results.
**Figure S1.** Risk of bias summary: review authors judgements about each methodological quality items of included RCTs.

## Data Availability

The data supporting the findings of this study are available from the corresponding authors upon reasonable request.
