# Supplementary material for: Biopsychosocial Approaches for the Management of Female Chronic Pelvic Pain: A Systematic Review
Source: BJOG. 2024 Oct 27;132(3):266–77. doi: 10.1111/1471-0528.17987 (PMC11704080; doi:10.1111/1471-0528.17987)
Supplement: Supplementary file 1 — Table S1. Biopsychosocial approaches used for chronic pelvic pain. Table S2. Characteristics of included studies. Table S3. Included studies reported outcome results. Figure S1. Risk of bias summary: review authors judgements about each methodological quality items of included RCTs. [file BJO-132-266-s001.docx]

**Supplementary materials:**

**Appendix S1: Example search strategy.**

Ovid MEDLINE(R)

1 exp Chronic Pain/

2 exp Pelvic Pain/

3 1 and 2

4 "chronic pelvic pain".ti,ab,kw.

5 "pelvic girdle pain".ti,ab,kw.

6 "perineal pain".ti,ab,kw.

7 "pudendal neuralgia".ti,ab,kw.

8 "vulva pain".ti,ab,kw.

9 vulvodynia.ti,ab,kw.

10 "painful bladder syndrome".ti,ab,kw.

11 "pelvic myalgia".ti,ab,kw.

12 (endometriosis adj3 pain).ti,ab,kw.

13 exp Endometriosis/

14 1 and 13

15 3 or 4 or 5 or 6 or 7 or 8 or 9 or 10 or 11 or 12 or 14

16 exp Models, Biopsychosocial/

17 biopsychosocial.ti,ab,kw.

18 exp self-management/

19 exp Pain management/

20 exp "Acceptance and Commitment Therapy"/ or exp Cognitive Behavioral Therapy/

21 exp Rehabilitation/ or rehabilitation.mp. or exp "Physical and Rehabilitation Medicine"/

22 "acceptance commitment therap*".ti,ab,kw.

23 "cognitive behavio* therap*".ti,ab,kw.

24 "pain management program*".ti,ab,kw.

25 "self management".ti,ab,kw.

26 "behavio* therap*".ti,ab,kw.

27 (cognitive adj2 therap*).ti,ab,kw.

28 mind body.ti,ab,kw.

29 holistic.ti,ab,kw.

30 patient cent*.ti,ab,kw.

31 "pain rehab*".ti,ab,kw.

32 psycholog*.ti,ab,kw.

33 "cognitive functional therap*".ti,ab,kw.

34 16 or 17 or 18 or 19 or 20 or 21 or 22 or 23 or 24 or 25 or 26 or 27 or 28 or 29 or 30 or 31 or 32 or 33

35 34 and 15

36 limit 35 to english language

**Table S1: Biopsychosocial Approaches used for Chronic pelvic pain.**

|  | Study | Population | Intervention | Treatment aim | Components of intervention | Freq/ intensity |
| --- | --- | --- | --- | --- | --- | --- |
| **Physio based treatments** | | | | | | |
| 1 | Rodrıguez-Torres^25^  2020 | CPP | Individualised rehabilitation programme.  Led by physiotherapist with 2 years + experience in CPP.  Physiotherapist with 2 years | Improving pain, functionality, postural control, and self-perceived health status. | 1. Identify goals, 2. Discuss strategies and give the patient op to make decisions considering preferences and interests, 3. Strategies for pain, posture, and function which included exercise and activity management and CPP education knowledge, 4. Analyse how well strategies work and revise until find a solution, evaluate impact after each session | 16 x 60 min sessions over 8 weeks |
| 2 | Ariza-Mateos^23^  2020 | CPP | Individualised patient-centred therapy intervention.  Led by a specialist therapist with education in pain management. | Improving quality of life, coping, pain severity, occupational performance, and physical activity. | 1. Identify workload-capacity difficulties (energy, time, attention), and functional limitations. 2. Information and self-management CPP education 3. Action plans for individualized goals factoring in workload- capacity. 4. Action plans evaluated and adjusted- highly painful activities were included but adjusted so could be performed without pain increase and exposure gradually increased. | 6 x 45 min sessions over 6 weeks |
| 3 | Ariza-Mateos^24^  2019 | CPP | Individualised graded exposure therapy +/_ manual therapy.  Delivered by graded exposure therapists and manual therapy therapists. | Reduction of fear-avoidance beliefs, pain intensity and interference with daily activities and function. | 1. Manual therapy- 20 mins soft tissue mobilisation, 15 mins deep pressure massage, 10 mins muscle energy techniques to strengthen and stretch.  2. Graded exposure: 5 tasks ranked as most fearful were selected for implementation, patients were gradually exposed to tasks which were adjusted so could be performed without pain increase and then progressed as fear reduced. | Manual therapy 2x week x 45 mins x 6 weeks for both intervention groups.  Graded exposure= additional 45 min session 1x week |
| 4 | Nygaard ^26^  2020 | CPP | Group-based multimodal physiotherapy in hospital setting versus primary care physical therapy.  Delivered by physiotherapists. | Compare the efficacy of interventions | 1. Group therapy involves the biopsychosocial model, combined body awareness therapy, patient education, and cognitive approach of “acceptance and commitment therapy” in a group setting (appendix provides a detailed tidier table describing intervention).  2. Women's health physical therapy provided by academic competence and in consultation with the woman- this was found to include -pelvic floor training combined with general exercises and relaxation exercises for 30% and soft tissue treatment alone for 50%. | Group= 10x days plus further 2-day sessions at 3,6 and 12 months  Physio: median number of sessions =14 (IQR=29) |
| **Cognitive behavioural therapy (CBT) informed interventions - individualised** | | | | | | |
| 5 | Goldfinger^31^  2016 | PVD | Cognitive behavioural therapy or physiotherapy.  No description of discipline that delivered CBT. | Determine the effects of each treatment in addressing bio-sexual components of PVD. | 1. CBT: education, a collaborative reconceptualization of PVD, desensitisation exercises including self-exploration, communication skills, cognitive restructuring, deep breathing, relaxation exercises, PFM exercises and dilators for progressive vaginal penetration exercises.  2. Physio: Education, manual techniques, surface EMG, hip stretches, deep breathing, relaxation exercises, PFM exercises, and dilators for progressive vaginal penetration exercises. | 8x 1.5 hour 1:1 sessions and homework delivered over 8-24 weeks. |
| 6 | Masheb^27^  2009 | PVD | CBT versus non-behavioural psychotherapy  Delivered by doctoral-level research therapists experienced in interventions. | Evaluate the efficacy of CBT against non-behavioural psychotherapy | 1. CBT: involved three overlapping phases: ^1^orientation to a self-management approach, ^2^skills acquisition, and ^3^skills practice. Components included: behavioural components; gate-control, activity pacing, and goal setting, sex therapy, cognitive components; identification of negative mood states, triggers, distortions and restructuring, relaxation components; diaphragmatic breathing and progressive muscle relaxation.  2. Non-behavioural psychotherapy: assisting patients in expressing their feelings whilst not making specific suggestions on how they should change. Therapists were trained to provide 1- a positive accepting attitude, 2. Engage in empathetic understanding, 3. To mirror to communicate understanding. | 60 min x 1x week, x 10 weeks |
| **CBT Group based interventions** | | | | | | |
| 7 | Bergeron^28^  2021 | PVD | Couples CBT versus topical lidocaine  CBT was delivered by Ph. Ph.D.-level clinical psychologists and supported by a manual. | Evaluate the efficacy of CBT against lidocaine. | CBT: Intervention included information about CBCT, education about PVD (impacts to sexuality and a multifactorial view of pain), breathing techniques, vaginal dilation exercises; cognitive diffusion, distraction with sexual imagery, expansion of sexual repertoire, and exercises to improve pain and sexuality-related couple interactions (e.g., communication).  2. 5% lidocaine ointment on vulva vestibule nightly x 12 weeks | 75 mins x 12 weeks vs lidocaine 5% x 12 weeks |
| 8 | Bergeron^29^  2001 | PVD | Vestibulectomy versus Surface EMG biofeedback versus Group based CBT.  EMG and GCBT delivered by Ph.D. level psychologists. | Evaluate and compare the efficacy of three interventions | 1. Vestibulectomy- minor day surgical procedure under general anaesthetic to excise of vestibular area to a depth of 2mm and a width of 1cm.  2. Biofeedback- training with sensor to reduce instability and hypertonicity. Protocol involved practice rest, rapid contractions, and endurance.  3. Group CBT treatment included: Education-PVD, multifactorial impact, sexual anatomy; progressive muscle relaxation; abdominal breathing; Kegel exercises; vaginal dilatation; distraction techniques focusing on sexual imagery; communication skills and cognitive restructuring. | 1. 1x day case procedure  2. 8 x 45 min sessions x12 weeks and portable home trainer to practice 2x day x 12 weeks.  3. 2hours x 8 sessions over 12 weeks. |
| 9 | Guillet^32^  2019 | PVD | Mindfulness-based Group CBT (MBCT) versus education group.  MBCT delivered by psychiatrist and education group meetings facilitated by psychiatric nurse practitioner | Compare the efficacy between interventions | 1. M-gCBT: Content involved CBT, sex therapy, mindfulness-based therapy, group therapy, educational videos standardised between both groups, daily homework, and practice of prescribed mindfulness exercises.  2. Education: modules regarding vestibule skin pain, sore pelvic muscles, and psychological dysfunction. Videos that were standardised between groups. | M-gCBT 2.5 hrs x 1 per week x 8 weeks.  Education group: weekly online education modules x 8weeks with 3x 1.5hr meetings. |
| 10 | Brotto  2019 | PVD | MBCT versus CBT group therapy.  Both delivered by therapists training in group therapy and experience in diagnosis and management of PVD. MBCT had additional mindfulness training. | Compare the efficacy between interventions | 1. CBT: Included PVD psychoeducation covering sexual desire, motivation, function, and mood. Behavioural skills training (eg, progressive muscle relaxation, diaphragmatic breathing, vaginal inserts, or challenging avoidance behaviour); cognitive restructuring and communication skills training. Facilitators and patients were supported by a manual that contained detailed descriptions of the exercises, diaries, and places to document homework activities.  2.MBCT: Included same components as CBT but additionally included different mindfulness meditations, specific exercises where vestibular pain was provoked while observing sensations mindfully. Plus 20-45 mins daily practice using audio recordings. | 2.25hrs x weekly x 8 weeks |
| **Acceptance commitment therapy (ACT) interventions that included and described acceptance, diffusion, contact with the present moment, self as context, values and committed action** | | | | | | |
| 11 | Hess Engstrom^71^  2021 | PVD | Internet-based ACT  MDT delivered online courses supported by manual. | Investigate intervention effects on pain during intercourse. | Each module had a specific theme and comprised of information and exercises. Themes covered included: Introductory information about vulvodynia, pain and pelvic floor function, values, thoughts, relationships, and maintenance. | 6 modules delivered over 6 weeks |
| ^26^12 | Hansen^33^  2023 | CPP | Nonspecific psychology, and endometriosis specific ACT based psychology group sessions compared to waiting list control (WLC)  Delivered by 2 psychologists. | To test the effects of all treatment arms on CPP and QoL. | Both interventions included:  Shared patient education content that covered CPP, psych impact of pain, stress and anxiety, depression and grief, healthy diet and exs, relationships, identity and meaning, a good life with CPP.  1. MY-ENDO: Included mindfulness and acceptance-based exercises and yoga practices.  2. Nonspecific psychology: Included mindfulness control which was relaxation to music, yoga control which was gentle stretches. | Both interventions involved 3hrly group sessions x 10 weeks, plus 30-45 min home practice exercises 5-7 x week. |
| Mindfulness-based interventions that described mindfulness-based treatment but did not include all ACT core processes | | | | | | |
| 13 | Moreira^37^ 2022 | CPP | Brief mindfulness-based intervention versus usual care (hormonal therapy and analgesics). | To test the effects of bMBI | Group-based bMBI-psycho education, mindfulness meditations, acceptance, mind-body connection and connection to avoidance (response /reaction), habits and behaviours, challenging ADLS. | 4 weeks x 1.5 hrs + 20-30 mins home practice. |
| 14 | Crisp^38^  2023 | CPP | Mindfulness-based stress reduction (MBSR) or self-paced  healthy lifestyle (HL) group interventions. | Determine the effectiveness of both interventions | 1. MBSR- online training consisted of classic journal and written guidance with YouTube videos found on palousemindfulness.com with weekly homework assignments, progress reports, access to online support training, body/mind connection, psychoeducation, yoga, thoughts & distractions, responding and reacting.  2. HL included lifestyle modifications including diet and exercise programmes, diet and exercise targets and coaching. | 6-week course for both groups, taught content plus 30 mins practice 5x week. |

**Table S2: Characteristics of Included Studies.**

|  | Author, year | Setting & location | Sample and method | Interventions | Outcomes (domain) | Follow up | Study Findings |
| --- | --- | --- | --- | --- | --- | --- | --- |
|  | RCTs |  |  |  |  |  |  |
| 1. | Rodrıguez-Torres^21^  2020 | Gynaecological dept.  Spain | 38 CPP  Randomised to 2 groups. | 1. Control (n=19): Information leaflet  2: Physio led Individualised rehabilitation programme (n=19) | 1. Mini best (Physical function) 2. TUG (Physical function) 3. EQ5D (QoL) 4. BPI sev (Pain) 5. BPI Int (functionality) 6. ODI (functionality) | Pre,  Post,  3m. | Significant between group differences in favour of intervention post treatment and 3 months. |
| 2 | Ariza-Mateos^19^  2020 | Gynaecological dept.  Spain | 44 CPP  Randomised to 2 groups. | 1. Control (n=22): Information leaflet  2: Physio led patient centred individual intervention that includes workload-capacity balance (n=22). | 1. VAS (Pain)  2. EQ5D (QoL)  3. CSQ (Psychological function)  4. COPM (functionality)  5. IPAQ (functionality) | Pre,  Post | Significant between group differences in favour of intervention for all outcome domains. |
| 3 | Ariza-Mateos^20^  2019 | Gynaecological dept.  Spain | 49 CPP  Randomised to 3 groups. | 1. Control (n=17): Information leaflet  2. Graded exposure + manual therapy (n=16)  3. Manual therapy (n=16) | 1. FABQ (Psychological function)  2. BPI sev (Pain)  3. BPI Int (functionality)  4. ODI (functionality) | Pre,  Post,  3m | Graded exposure added to manual therapy is distinctly superior to manual therapy alone in maintaining improvements in all domains. |
| 4 | Nygaard ^22^  2020 | Pelvic floor centre in hospital setting and primary care physical therapy.  Norway | 62 CPP  Randomised to 2 arms. | 1. Intervention (n=26): Group- based multimodal physiotherapy in hospital setting.  2. Comparator (n=25): primary care physical therapy. | 1. Average NRS (Pain)  2.Standardized mensendieck test (Physical function)  3.TSK (Phycological function)  4. EQ5D (Quality of Life)  5. Hopkins symptom checklist (Psychological function)  6. Continence  7. Sexual function (Y/N to 3 questions) | Pre,  Post,  12m | The intervention group showed significantly better improvement in pain intensity, respiratory patterns and the TSK, no significant differences were observed between the groups for other measures. |
| 5 | Goldfinger^27^  2016 | Canada | 20 PVD  Randomised to 2 groups. | 1. CBT (n=10)  2. Physio (n=10) | 1. NRS during intercourse (Pain)  2. FSFI (Sexual function)  3. PCS (Psychological function)  4. CSQ (Psychological function) | Pre,  Post,  6m | CBT and PT were associated with clinical meaningful improvement in all outcomes and were maintained at 6 months, few between group differences were identified. |
| 6 | Masheb^23^  2009 | Healthcare providers or responded to advert.  USA | 50 PVD  Randomised to 2 groups. | 1. Control (n=25): Supportive psychotherapy  2. CBT (n=25) | 1.Pain speculum and swab test.  2. Severity subscale (Pain)  3. MPI (Pain)  4. FSFI (Sexual function)  5. BDI (Psychological function)  6.PASS (Psychological function)  7. Treatment credibility, satisfaction and GIPC. | Pre,  Post,  6 m  12 m | CBT demonstrated significantly greater changes in all outcome domains compared to supportive psychotherapy. |
| 7 | Bergeron^24^  2021 | Adverts in newspapers, universities, hospitals and medical clinics, and prior study participation Canada | 108 PVD randomised to two treatment arms. | 1. Control (n=53): topical lidocaine  2. Couple CBT (n=55) | 1.NRS during intercourse (Pain)  2. PASS (Psychological function)  3. FSFI (Sexual function)  4. FSDS (Sexual Function)  5. PCS (Psychological function)  6. Treatment credibility, satisfaction and GIPC. | Pre, Post,  6 m. | CBT yielded significantly more improvements than lidocaine in pain, pain anxiety and pain catastrophising post treatment and at 6 months. |
| 8 | Bergeron^25^  2001 | Media announcements and professional referral.  Canada | 78 PVD  Randomised to 3 arms. | 1. CBT (n=26)  2. Surface EMG (n=26)  3. Vestibulectomy (n=26) | 1.NRS during intercourse (Pain)  2. MPQ-Pain rating index (Pain)  3. MPQ-sensory scale (pain)  4.Sexual history form – Global sexual functioning score (Sexual function)  5. DSFI- information subscale (Sexual function)  6.BSI- global severity index (Psychological function) | Pre, Post,  6 m. | All 3 groups significantly improved on measures of psychological adjustment and sexual function and pain measures at post treatment and 6-month FU. Vestibulectomy was superior on pain measures however 7 women assigned did not go ahead with treatment. |
| 9 | Guillet^28^  2019 | Programme of vulva health  USA | 31 PVD randomised to 2 arms | 1. Control (n=17): Education support group.  2. Intervention (n=14): Mindfulness- based Group CBT (MBCT) | 1. Tampon test (Pain)  2. PCS (Psychological function)  3. FSDS (Sexual function)  4. FSFI (Sexual function)  5. GAD-7 (Psychological function)  6. BDI (Psychological function) | Pre,  Post  6m | Both treatments were effective in reducing pain and distress. MBCT showed statistically significant improvements in FSFI, GAD-7 and BDI compared to control and therefore may offer some advantage in reducing associated distress. |
| 10 | Brotto^26^  2019 | Tertiary sexual health centres  Canada | 130 PVD  Randomised to 2 arms | 1.CBT (n=63)  2. MBCT (n=67) | 1.Pain during intimacy (Pain)  2.vulvavagsiometer (Pain)  3.FSFI (Sexual function)  4.FSDR (Sexual function)  5.PCS (Psychological function)  6.PVAQ (Psychological function)  7.CPAQ (Psychological function)  8. GIPC/ satisfaction | Pre,  Post,  6m | There was a significant interaction between group and time for self-reported pain, such that improvements with MBCT were greater than those with CBT. For all other endpoints, both groups led to similar significant improvements, and benefits were maintained at 6 months. |
| 11 | Hess Engstrom^60^  2021 | Gynaecology clinics  Sweden | 99 PVD  Randomised to 2 groups. | 1.Control (n=47): WLC  2. Internet based treatment using ACT (n=52) | 1.NRS during intercourse (Pain)  2. Impact on sexual function % scores (Sexual Function)  3. CPAQ (Psychological function) | Pre,  Post  10m | Treatment was associated in significant changes for pain during intercourse and pain acceptance. High dropout was a large study limitation. |
| 12 | Hansen^29^  2023 | Outpatient endometriosis clinics  Denmark | 58 CPP  Randomised to 3 arms | 1. Specific mindfulness and acceptance-based intervention (MY-ENDO) (n=20)  2. Nonspecific psychological intervention (n=19)  3. Control: WLC (n=19) | 1. NRS (Pain)  2.EHP-30 (Quality of life)  3.WAI (functionality)  4.CPAQ (psychological function) | Pre  Post- 12 wk | Compared to WLC, both psychological interventions did not significantly reduce pain. Both psychological interventions did significantly improve QoL subscales. MY-ENDO was not superior to nonspecific psychological intervention. |
| 13 | Moreira^33^  2022 | Outpatient clinics  Brazil | 63 CPP  Randomised to 2 arms | 1. brief Mindfulness-based intervention (bMBI) (n=31)  2. Usual care (n=32) | 1. NRS (Pain)  2. Perceived stress scale (PSS)  3.SF-36 (Quality of life)  4. Five facets of mindfulness questionnaire (FFMQ) | Pre  Post  8 wks | The results show that bMBI significantly improved pain unpleasantness, pelvic pain,  and dyschezia immediately post-treatment  and decreased all endometriosis-related pain after the follow-up but effect size for QoL measure domains was < small |
| 14 | Crisp^34^  2023 | Military treatment facility  USA | 41 CPP | 1. Mindfulness-based stress reduction (MBSR) (n=21)  2. Healthy Lifestyle group intervention (n=20) | 1. BPI (pain)  2. Patient health Questionnaire (PHQ9)  3.FFMQ  4. Cytokine concentration | Pre  Post | MBSR or HL had reduced depression scores and altered circulating cytokine levels, however only those receiving MBSR had reduced pain perception. |

**Figure S1: Risk of Bias Summary: Review Authors Judgments About Each Methodological Quality Items of Included RCTs.**

**
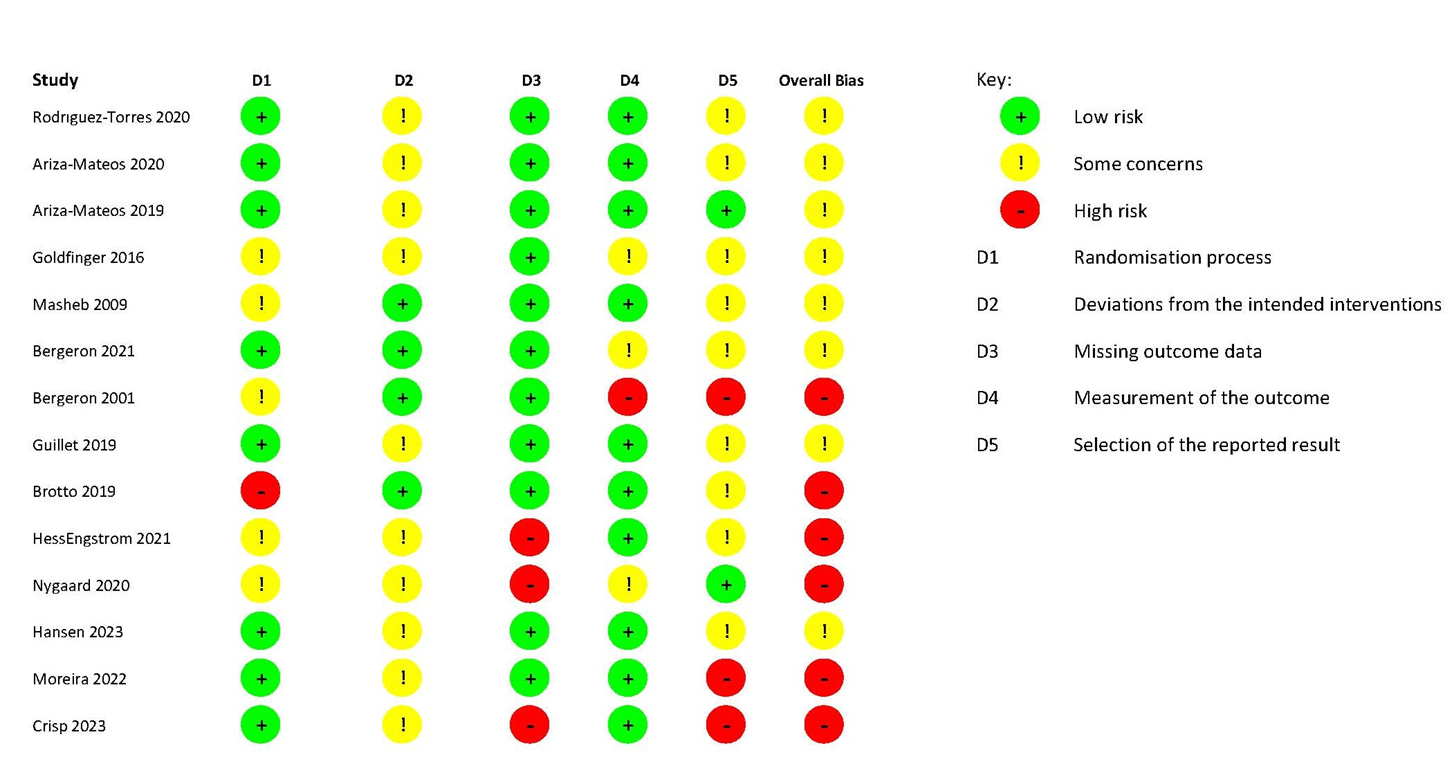
**

**Figure S1 Legend- Risk of Bias Explanation**

***Randomisation process***

5/14 studies were considered to have some bias either due to insufficient detail describing the randomisation process or baseline differences in potential confounding variables between groups^22,23,25,27^. One study was considered high bias as 63.9% of patients were non-randomly assigned to a group^26^.

***Deviations from intended intervention***

Participants and persons delivering the intervention where unblinded to intervention. Expectation of benefit can affect intervention compliance. We judged studies for their assessment of treatment expectation. Low ROB was considered if there were no significant differences between treatment arms on expectation (4/14 studies) ^23–26^ and some concerns if there were differences between treatment arms or if no assessment of expectation was made (10/14 studies). ^24–27^

***Missing outcome data***

Three studies were considered high bias, 2 reported high attrition rates which were excluded from efficacy analysis^22,30^, and one did not describe how missing data was handled ^34^.

***Measurement of outcome***

High risk of bias was considered when assessors of outcome were not blinded^25^, whereas some bias was described when this was not clear^22,24,27^.

***Selection of reported result***

Some bias was considered where; studies had not used a pre-registered protocol (8/14)^21,23–25,27,29,33,34^, no time reference point was considered for pain outcomes (4/14)^23,24,27,30^ or change in sub-scales of a measure were reported rather than the composite score (4/14)^19,21,26,28^. High bias was considered when studies used only part of a validated outcome rather than the complete measure or baseline scores were not reported^25,33,34^.

**Appendix S2: Descriptive comparison of absolute treatment effects where comparable outcomes were used.**

**Physio Led Interventions**

Change in terms of QoL was comparable between the cumulative-complexity model of care ^19^ (EQ5D VAS: pre 53.44 (14.91)- post 70.06 (16.44)) and ICPR ^21^ at baseline and post treatment (pre-53.33(25.24)- post 70.59 (19.91)- 3-month FU 61.65 (18.59)). For the cumulative-complexity model of care only post treatment outcomes were recorded and therefore it is not possible to conclude whether benefits were maintained after treatment. It should also be noted the treatment duration for both studies were not comparable (16 x60 min sessions over 8 weeks^21^ vs 6 x 45 min sessions over 6 weeks^19^).

In comparison greater improvement was seen with the intervention GET+MT intervention^20^ when compared to ICPR intervention study results^21^ for outcomes of; **pain severity** (BPI) ICPR pre: 6.01 (1.95)- post 3.6 (1.55)- FU 5.52 (1.19) vs GET+MT pre: 6.01 (1.95)- post 4.33 (2.16)- FU 3.26 (1.97), **pain interference** BPI ICPR pre: 5.13 (2.39)- post 3.26 (2.33)- FU 4.48 (2.34) vs GET+MT pre: 5.09 (2.45)- post 3.33 (1.66)- FU 2.64 (2.41), **disability** (ODI) pre: 33.95 (18.16)- post 14.74 (8.59)- FU 26.61 (4.31) vs GET+MT pre: 29.63 (13.74)- post 17.67 (7.69)- FU 7.33 (5.85). However, the duration of both treatments was not comparable, and the Rodriguez study population had a significantly shorter pain duration although other variables were comparable at baseline.

**Individualised CBT**

In both studies, the only comparable measure was change in sexual function using the 19 item FSFI scale (score range 2-36). Comparable change for the CBT intervention in both studies was observed post treatment. Greater change at FU was observed within the Goldfinger study were CBT treatment also included pelvic floor muscle (PFM) exercises, and dilators for progressive vaginal penetration (FSFI Goldfinger: pre- post change 5.96, pre- FU change 8.29, Masheb: pre- post change 6.1, pre- FU change 6.7). The differing control interventions and sample sizes limits any comparison in relation to the relative effects of each intervention.

**Group Based CBT**

Comparable change in PCS was observed in ¾ studies following CBT group-based interventions (PCS Brotto^26^: pre- post change -13.05 CBT, -11.28 MBCT, pre- FU change -14.79 CBT, -15.37 MBCT; Guillet^28^: pre- post change -10.68, pre- FU change -12.24; Bergeron^24^: pre- post change -14.85, pre- FU change -14.95).

Three studies used the FSFI. Brotto and Bergeron^24,26^ used the 19 item FSFI (score range 2-36), whilst Guillet^28^ reports a score range of 2-95. Where a score range of 2-36 is used, the addition of mindfulness to CBT was associated with a slightly higher degree of change than CBT alone, whereas there was no difference between couples-based CBT and CBT alone (FSFI Brotto^26^: pre- post change 1.61 CBT, 2.22 MBCT, pre- FU change 2.02 CBT, 5.18 MBCT; Bergeron^24^: pre- post change 2.07, pre- FU change 1.79. The degree of FSFI change reported in group-based CBT was smaller than the individualised CBT therapies.

Where the FSDS questionnaire was used to measure sexual distress and desire- change was slightly higher for CCBT post intervention compared to MBCT and CBT but comparable at follow up for all interventions (FSDS Brotto^26^: pre- post change 10.75 CBT, 7.68 MBCT, pre- FU change -11.91 CBT, -11.99 MBCT; Guillet^28^: pre- post change -13.5, pre- FU change -13.01; Bergeron^24^: pre- post change -14.85, pre- FU change -10.95).

**ACT Based Therapies**

Two studies measured change in the chronic pain acceptance questionnaire (CPAQ). Higher post treatment change was reported in the face to face delivered ACT intervention compared to the internet-based intervention (CPAQ Hess Engstrom: pre-post change 6.62, pre-FU change 12.18; Hansen pre-post change 13.71).

It should be noted that despite use of similar outcome domains treatment durations the control comparators where very different between studies. Variations in outcome parameters used across all studies prevented further narrative comparison.

**Table S3:** **Included Studies Reported Outcome Results**

| Study | Pop | Baseline  (Age/pain duration yrs) | Outcomes (domain) | Baseline for intervention group/(s) | Post Rx mean(s) | LT FU Mean (s) |
| --- | --- | --- | --- | --- | --- | --- |
| **1:1 Physio** | | | | | | |
| Rodrıguez-Torres^21^  2020 | CPP | Age:18-80, mean 42.93 (8.22)  Pain dur:  2.5 (12.03) | 1. Mini best (Physical)  2. TUG (Physical)  3. EQ5D VAS (QoL)  4. BPI sev (Pain)  5. BPI Int (function)  6. ODI (function) | 1. 15.92 (3.29)  2. Dual 15.54 (2.28)  3. 53.33 (25.24)  4. 6.01 (1.95)  5. 5.13 (2.39)  6. 33.95 (18.16)  **Note: *cumulative-complexity model n=22*** | 1.18.93 (2.75)**  2. Dual 13.21 (2.8)*  3. 70.59 (19.91)*  4. 3.6 (1.55)**  5. 3.26 (2.33)*  6. 14.74 (8.59)** | 1.16.45 (1.53)  2. 14.42 (2.54)  3. 61.65 (18.59)  4. 5.52 (1.19)*  5. 4.48 (2.34)  6. 26.61 (4.31)** |
| Ariza-Mateos^19^  2020 | CPP | Age:18-65, mean 42.62 (8.57)  Pain dur:  not stated. | 1. VAS (Pain)  2. EQ5D VAS (QoL)  3. CSQ (Psychological)  4. COPM (function) performance  4. COPM (function) Satisfaction  5. IPAQ (function) | 1. 5.39 (1.95)  2. 53.44 (14.91)  3. No total score  4. Perform: 4.04 (1.29)  4. Satis: 4.67 (1.65)  5. 1563.65 (918.15)  ***Note: ICPR n=18*** | 1. 2.21(1.81)*  2. 70.06 (16.44) *  3. No total score  4. Perform: 6.97 (1.73) *  4. Satis: 7.28 (1.7) *  5. 2248.53(1145.21) | n/a |
| Ariza-Mateos^20^  2019 | CPP | Age:18-65, mean 42.26 (9.57)  Pain dur:  9 (7.81). | 1. FABQ (Psychological)  2. BPI sev (Pain)  3. BPI Int (function)  4. ODI (function) | 1. 18.74 (2.75)/ 19.39 (2.4)  2. 6.01 (1.95)/ 5.83 (2.02)  3. 5.09 (2.45)/6.48 (1.49)  4. 29.63 (13.74)/31.40 (8.17)  ***Note: GET+MT n=16/ MT n=16.*** | 1. 10.41(7.61)*/14.83 (4.23)*  2. 4.33 (2.16)*/ 4.5(1.78)  3. 3.33 (1.66)*/ 5.06(1.53)  4. 17.67(7.69)*/21.82(12.02) | 1.6.37(7.54)*/13.44(6.36)*  2. 3.26(1.97)*/4.08(1.16)*  3.2.64(2.41)*/ 4.08(1.16)*  4.7.33(5.84)*/11.92(6.71)* |
| Nygaard ^22^  2020 | CPP | Age: 20-65  Mean 39.7 (10.9)  Pain dur:  12 (37%) | 1. Average NRS (Pain)  2.TSK (Psychological)  3. EQ5D (Quality of Life)  4. Hopkins symptom checklist (Psychological) | 1.4.8 (2)/4.5 (2.8)  2. 24.4(4.8)/23(6.3)  3. 58(19.1)/58.2(22.5)  4. 1.83(0.45)/1.78(0.51)  **Note: ACT physio n=26/ primary care physio n=25** | 1.3.7(2.0)/3.7(2.4) | 1.3.0(2.4)*/4.0(2.9)  2.19.4(4.3)*/20.8(5.9)  3.62.1(20.3)/64.2(18.1)  4.1.52(0.38)/1.64(0.54)  Significance values reflect between group changes – within group not stated. |
| **CBT 1:1** | | | | | | |
| Goldfinger^27^  2016 | PVD | Age: 18 +  Mean 27.40 (11.05)  Pain Dur:  4.36 (2.87) | 1. NRS intimacy (Pain)  2. FSFI (Sexual)  3. PCS (Psychological)  4. CSQ (Psychological) | 1. 5.20 (1.40)/ 5.05 (1.86)  2. 22.26 (5.57)/22.59(7.20)  3. 21.40 (7.11)/15.22 (12.17)  4. 2.56 (0.88)/2.33 (1.58)  ***Note: CBT n=10/Physio n=10*** | 1.2.6 (1.43) */ 2.7(2.36) **  2.27.37(4.61)/ 27.06 (4.25)  3.10.40 (7.89)*/ 7.89 (7.80)**  4.4.33(1.0)**/4.22(1.09)* | 1.2.10(1.37) **/2.4(2.63) **  2.29.69 (5.12)*/ 24.29 (7.18)  3.9.10 (8.27)**/8.33(10.14)**  4.4.33 (1.22)*/4.44(1.33)* |
| Masheb^23^  2009 | PVD | Age: 21 +  Mean 43 (12.1)  Pain Dur:  8.4 (7.8) | 1. MPI (0-6) (Pain)  2. FSFI (Sexual)  3. BDI (Psychological)  4. PASS (Psychological) | 1. 2.6(1.2)/ 3.0 (1.3)  2. 15.9 (7.7)/18.4 (7.5)  3. 12.1 (10.2)/12.5 (8.1)  4. 72.6 (35.3)/73.0 (33.9)  ***Note: CBT n=25/ supportive psych n=25*** | 1. 1.6 (0.3)* / 1.9 (0.2)*  2. 22.1 (2.2)*/ 19.5 (2.2)  3. 9.9 (2.0)/9.9 (1.9)  4. 64.2 (7.2)*/62.8 (6.9)* | 1. 1.3 (0.3)*/1.3(0.3)*  2. 22.6 (2.2)/18.6 (2.2)  3. 7.3 (2.0)/ 11.5 (1.9)  4. 55.3 (7.2)*/ 65.2 (6.9)* |
| **CBT Group** | | | | | | |
| Bergeron^24^  2021 | PVD | Age: 18-45  Mean 27 (6.26)  Pain dur:  6.52 (5.2) | 1.NRS intimacy (Pain)  2. PASS (Psychological)  3. FSFI (Sexual)  4. FSDS (Sexual)  5. PCS (Psychological) | 1.6.81 (1.77)/6.51 (1.82)  2.41.75(13.36)/37.58(16.35)  3.17.30(5.02)/16.96 (4.53)  4. 34.64(9.40)/33.5 (10.15)  5.28.04(9.96)/25.58(10.57)  ***Note: CBT n= 55/Lidocaine n=53*** | 1. 4.70 (2.21)**/ 4.67 (2.29)**  2.28.5 (12)**/ 32.42 (17.79)  3.19.37(5.27)**/18.84(5.47)*  4.21.63(12.9)**/28.37 (14.56)*  5.13.15(8.42)**/18.25(13.19)** | 1. 4.45 (2.51**)/ 4.7 (2.58)**  2.25.89(14.75)**/27.64 (19.58)**  3.19.09(5.16)/19.61(5.42)  4. 23.69(14.47)/24.32(14.82)  5.13.09(11.18)**/15.24(13.81)** |
| Bergeron^25^  2001 | PVD | Age: 18-50  Mean 26.8 (5.4)  Pain dur:  4.78 (4.12) | 1.NRS during intercourse (Pain)  2. MPQ-Pain rating index (Pain)  3. MPQ-sensory scale (pain)  4.Sexual history form – Global score (Sexual)  5. DSFI- information subscale (Sexual)  6.BSI- global severity index (Psychological) | 1.7.18(1.62)/6.93(1.8)/7.14(1.53)  2.26.82(14.68)/26.46(15.99)/28.93(12.29)  3.17.86(8.4)/17.07(8.34)/18.61(7.28)  4.0.47(0.11)/0.51(0.11)/0.51(0.13)  5.21.68(1.91)/21.46(2.33)/21.82(2.31)  6.53.32(9.62)/54.11(8.78)/56.36(8.11)  ***Note: vestibulectomy n=26/EMG n=26/CCBT n=26*** | 1.3.93(3.25)**/5.43(2.36)*/6(2.13)*  2.15.86(16.18)**/23.79(17.23)*/27.75(15.09)*  3.10.82(9.74)**/15.57(10.18)*/18.68(8.69)  4.0.49(0.14)/0.51(0.08)/0.49(0.12)  5.22.41(1.74)/22.18(1.61)/21.75(2.15)  6.52(8.25)/51.29(8.93)/52.89(7.21) | 1.3.41(3.17)**/4.5(2.63)**/4.46(2.47)**  2.14.27(13.06)**/20.43(18.1)**/20.93(14.18)**  3.9.45(8.19)**,13.82(10.66)**/14.75(8.87)**  4.0.45(0.15)*/0.48(0.08)*/0.48(0.11)*  5.22.46(1.9)*/23.36(1.81)*/22.25(1.84)*  6.50.09(10.49)*/50.79(9.39)*/51.79(7.61)* |
| Guillet^28^  2019 | PVD | Age:18-55  Mean 31.6 (6.6)  Pain dur: NS | 1. Tampon test (Pain)  2. PCS (Psychological)  3. FSDS (Sexual)  4. FSFI (Sexual)  5. GAD-7 (Psychological)  6. BDI (Psychological) | **No pre/post scores reported results provided are within group changes  ***Note: group education classes n=17/MBCT n=14*** | 1.-1.35*/-1.33*  2.-7.72*/-10.68**  3.-4.87*/-13.5**  4.2.59/15.09 **  5.0.40/-2.98 **  6.-1.74/-3.79 * | 1.-1.88 **/-2.4 **  2.-11.16**/-12.24**  3.-10.52**/-14.53**  4.1.23/13.33*  5.-0.26/-3.10**  6.-0.02/-5.03* |
| Brotto  2019^26^ | PVD | Age:19+  Mean 32.35 (8.21)  Pain dur: 7.9 (6.67 | 1.Pain during intimacy (Pain)  2.vulvavagsiometer (Pain)  3.FSFI (Sexual function)  4.FSDR (Sexual function)  5.PCS (Psychological function)  6.PVAQ (Psychological function)  7.CPAQ (Psychological function) | 1.5.86(2.13)/6.69(1.91)  2.6.62 (2.19)/6.66(2.17)  3.21.18 (6.10)/19.57(6.29)  4.35.68(10.43)/34.28(11.18)  5.25.62(11.97)/26.92(12.86)  6.42.46(12.12)/43.24(14.67)  7. Total score not stated  ***Note CBT n=67/MBCT n=63*** | 1.4.65(2.21)/4.34(2.22)  2. 3.60(2.14)*/ 3.21(1.96)*  3. 23.41(5.72)*/21.79(6.83)*  4.24.93(9.31)*/26.60(13.35)*  5.12.57(8.13)*/15.64 (12.54)*  6. 40.32 (14.19)*/39.74(13.72)*  7. Total score not stated | 1.4.03(2.11)/3.39(1.89) *  2. 2.86 (1.89)*/2.92(2.31)*  3.23.20(5.45)*/24.75(5.62)*  4. 23.77(9.61)*/22.81(12.98)*  5.10.83 (7.66)*/ 11.55(9.91)*  6. 38.76(14.47)*/38.50(13.88)*  7. Total score not stated |
| ACT | | | | | | |
| Hess Engstrom ^60^  2021 | PVD | Age: 18 +  Mean 24.5 (4.4)  Pain Dur:  4.9 (4.2) | 1.NRS intimacy (Pain)  2. CPAQ (Psychological) | 1.6.85 (2.37) n=49  2.62.63 (15.33) n=48  ***Note Internet-based ACT n=52 at enrolment*** | 1.3.33 (2.81)*n=32  2. 69.25 (12.07)* n=32 | 1. 3.70 (2.16)* n=27  2. 74.81 (16.42)* n=26 |
| Nygaard ^22^  2020 | See physio above | | | | | |
| Hansen^29^  2023 | CPP | Age:18-47  Mean 31.4 (7.76)  Pain dur: 16.3 (7.15) | 1. NRS (Pain)  2.EHP-30 (QoL)  3.WAI (functional)  4.CPAQ (psychological) | 1. 6.11 (2.05)/ 5.53(1.90)  2. Total score not provided  3. 32.70(9.95)/35.36( 7.16)  4. 52.15 (19.35)/53.33(12.92)  **Note: MY-ENDO n=20 /Nonspecific psychology n=19** | 1. Mean and SD not reported- non significant change reported  2. Total score NS-subscales control/powerless/emotional wellbeing & social*  3. 36.94 (8.8)*/35.95 (7.34)  4. 65.86 (20.66)*/64.62(13.5)* |  |
| Mindfulness-based interventions | | | | | | |
| Moreira^33^  2022 | CPP | Age: 18-50  Mean  34.7 (8.1)  Pain dur:  Not stated | 1. NRS (Pain)  2. Perceived stress scale (PSS)  3.SF-36 (QoL)  4. Five facets of mindfulness questionnaire (FFMQ) | 1. 8.50 [7.25,10.00]  2. 26.00 [22.25, 30.75]  3. Separate items vs total scores reported  4. 115.60 [18.54]  **Median and q1, q3 (in Brackets)**  **Note: bMBI n=31** | 1. 5.00 [3.00,8.00]  2. 22.00 [19.00, 26.75]  3. Separate items vs total scores reported* for mental health subscale  4. 125.42 [17.39] | 1. 5.00 [2.25,6.00]  2. 21.00 [18.25, 25.25]  3. Separate items vs total scores reported* for mental health subscale  4. 123.95 [23.12] |
| Crisp^34^  2023 | CPP | Age: MBSR 27-32, HL 39-44  Pain dur:  Not stated | 1. BPI (pain)  2. BPI Int  3. Patient health Questionnaire (PHQ9)  4.FFMQ  5. Cytokine concentration | Baseline averages are not stated for any measure except for in graph format.  **Note: MBSR n=21/Healthy lifestyle n=20** | 1. No diff between groups at any time point  2. -3.0 (-1.0)**/ -3.0 (-2.0)  3.-4.0 (0.0)*/ -2.0* (0.0)  4. Raw data or change scores not reported  5. Significant change stated but p value and CI not reported. |  |
| Key: *=P<0.05, **p<0.001 | | | | | | |
